# Salivary flow rate and the risk of cognitive impairment among Korean elders: a cross-sectional study

**DOI:** 10.1186/s12877-021-02200-2

**Published:** 2021-04-14

**Authors:** Minh-Tung Do, Huong Vu, Jong-Koo Lee, Sang-Min Park, Joung-Sik Son, Hyun-Duck Kim

**Affiliations:** 1 Department of Urology, Seoul National University College of Medicine, Seoul, 03080 Republic of Korea; 2 Department of Preventive and Social Dentistry, School of Dentistry, Seoul National University Seoul, 101, Daehak-ro, Jongno-gu, Seoul, 03080 Republic of Korea; 3 Department of Family Medicine, Seoul National University College of Medicine, Seoul, 03080 Republic of Korea; 4 Department of Family Medicine, Korea University Guro Hospital, 148 Gurodong-ro, Guro-gu, Seoul, 08308 South Korea; 5 Dental Research Institute, Seoul National University, Seoul, 03080 Republic of Korea

**Keywords:** Salivary flow rate, Cognitive impairment, Epidemiology, Elder, Korean

## Abstract

**Background:**

Salivary function has been suggested to be associated with cognitive impairment. However, the effect of salivary flow rate (SFR) on cognitive impairment remains unclear. This study aimed to investigate whether SFR is associated with cognitive impairment among Korean elders.

**Methods:**

This cross-sectional study included 649 elders aged 65 and older in the Korean community-dwelling population. Cognitive impairment was assessed using the Mini-Mental Status Examination. Unstimulated SFR was measured and dichotomized. Denture status, age, sex, education level, smoking, drinking, diabetes, hypertension, and obesity were considered confounders. Multivariable logistic regression analysis was applied to assess the adjusted association. Stratified analysis by sex and denture status was performed to clarify the effect modification.

**Results:**

Participants without cognitive impairment showed a higher SFR level than those with cognitive impairment (0.81 mL/min for non-cognitive impairment versus 0.52 mL/min for cognitive impairment, *p <* 0.001). After controlling for confounders, participants with low SFR (< 0.3 mL/min) were more likely to have cognitive impairment by 1.5 times than participants with normal SFR (odds ratio [OR] = 1.5, confidence interval [CI] = 1.05–2.10). The association of low SFR with cognitive impairment was higher in women and dentate participants: about 10% higher in women (OR = 1.63, CI = 1.07–2.50) and about 22% higher in dentate participants (OR = 1.82, CI = 1.41–2.90).

**Conclusions:**

Salivary flow rate is independently associated with cognitive impairment among Korean elders. The association was modified in females and dentate elders. Physicians and dentists should consider low SFR and cognitive impairment as a risk factor between them in clinics.

**Supplementary Information:**

The online version contains supplementary material available at 10.1186/s12877-021-02200-2.

## Background

Cognitive impairment in the older population has been a head-aching global health problem due to its unclear mechanism and complicated relationship with aging-related common disorders. The prevalence of dementia in people aged 60 and over is about 5–7% in most world regions [1]. The number of dementia was estimated at 50 million worldwide in 2018, and it was predicted being triple in 2050 [2]. Korean population has gained the fastest aging globally and was expected to be a super-aged society in the next 5 years. The prevalence of mild cognitive impairment among Korean elders was estimated to be as high as 24.1%, which would be a severe public health issue [3]. Thus, it is crucial to unmask the risk factors of this disorder screening and prevention.

Cognitive impairment has been associated with various oral health problems in late adulthood [4]; the poorer oral health is more likely to diminish cognitive function. Compared to those without cognitive impairment, older people with cognitive impairment have more likelihood of experiencing periodontitis [5], masticatory problems [6], and likely to have a higher number of lost tooth [7] and non-rehabilitated lost teeth [8]. However, the association of salivary flow rate and cognitive impairment has not been well investigated. Normal salivary secretion is essential in maintaining efficient mastication and other oral functions [9–11]. Salivary secretion is controlled by the autonomic nervous system and regulated by reflex pathways, including the salivation center in the brain [9]. The degeneration of the central nervous system in cognitive impairment could alter the afferent or efferent reflex, decreasing the salivary flow rate (SFR). The autonomic dysfunctions observed in cognitive impairment may also contribute to hyposalivation [12]. However, only two papers have reported the relationship between cognitive impairment and SFR [13, 14]. Moreover, the relationship between cognitive impairment and SFR has not been reported among Koreans.

A Danish study reported that men with a decline in cognitive performance had a significantly lower SFR than the controls [13]. Patients with an early stage of dementia also show a selective impairment in submandibular gland function [14]. However, no study has considered the relationship after controlling potential confounders such as socio-demographic factors, general health problems and smoking, behaviors and denture status.

Hence, we hypothesized that SFR was associated with cognitive impairment after controlling for various confounders including denture status, socio-demographic factors [15] such as age, sex and education level, behaviors [15, 16] such as smoking and drinking, and general health problems [17–19] such as diabetes, hypertension and obesity. This cross-sectional study aimed to evaluate the adjusted association of SFR with cognitive impairment among Korean elders and its effect modification by sex and denture status.

## Methods

### Ethical considerations and study design

This study was approved by the Institutional Review Board for Human Subjects at the Seoul National University School of Dentistry and Seoul National University College of Medicine (approval number: S-020190017 and C-1803-117-932). All participants provided written, informed consent of their record. This study was the baseline (2018–2019) of the community health education cohort, which combined medical and dental health. After several weeks of the advertising period which was performed in advance of the survey, participants were recruited. The survey was conducted at a community health center in Songuk-Gu, Seoul. Systemic health status and oral health status were assessed by trained medical and dental health professionals in the project who received calibration training beforehand.

### Study population

The inclusion criteria were as follows: 1) community-dwelling people aged 65 and above who lived in Songbuk-gu, 2) elders without critical diseases encompassing cancer, paralysis, stroke, and cardiovascular diseases (angina pectoris, myocardial ischemia, or heart failure), 3) no problem and willing to follow the recommendation of the cohort procedures, 4) voluntarily joined with self-written informed consent, and 5) without any missing information for this study. The 743 elders in Songbuk-gu were total voluntary participants in this study who were recruited according to the recruit guideline. They completed the health assessment and questionnaires. After excluding 94 participants with incomplete information, 649 elders were included in the final analysis.

### Assessment of cognitive impairment

The Mini-Mental State Exam (MMSE) is a widely used screening tool for cognitive function [20]. The Korean version (MMSE-KC) was developed as a part of the Korean version of the Consortium to Establish a Registry for Alzheimer’s Disease Assessment Packet [21]. The MMSE-KC contains 19 items adding up to 30 points (10 points for orientation, 6 points for verbal memory, 5 points for concentration and calculation, 5 points for language, 3 points for praxis, 1 point for visuospatial construction), with higher scores indicating better cognitive performance. Because of the high prevalence of illiteracy in elderly Koreans, two items focusing on judgment ability replaced the reading and writing items of the original version of MMSE in the MMSE-KC. The MMSE-KC showed adequate diagnostic accuracy for moderate dementia, with an area under the receiver operating characteristic curve of approximately 0.9. The total score was used to determine cognitive impairment (≤ 23 points) and non-cognitive impairment (> 23 points) according to the previous studies [22, 23].

### Unstimulated salivary flow rate measurement

Participants were advised not to eat or drink (except for water) about 8 h before the procedure in the morning in March (Spring) and September (Autumn). When coming to the test office, they were instructed to rinse their mouth with distilled water and take a rest for several minutes. The participants were instructed to swallow once before measurements began, then to keep on drooling for five minutes into a tube with previous weight measurement. They were also advised to minimize the movement of their mouth and not swallow any saliva during the procedure. The collected saliva was weighed and converted to volume (1:1 from grams to milliliters). The SFR (mL/min) was calculated by dividing the volume by time. Although previous studies [13, 24, 25] adopted hyposalivation (SFR <  0.1 mL/min), Dawes et al. [26] suggested low salivation (SFR < 0.3 mL/min). Since our data showed small numbers in cognitive impairment with hyposalivation (*n* = 31), we dichotomized SFR according to the suggestion of Dawes: normal SFR (≥ 0.3 mL/min) and low SFR (< 0.3 mL/min) [26].

### Assessment of confounders

According to previous reports, confounders in this study included denture status, socio-demographic factors [15] such as age, sex and education level, behaviors [15, 16] such as smoking and drinking, and general health problems [17–19] such as diabetes, hypertension and obesity.

Participants were interviewed face-to-face by trained interviewers for information regarding socio-demographic and behavioral factors. Interviewers were recruited from the survey area and trained before the main survey using structured questionnaires. Socio-demographic factors included education level, age, and sex; health-related behavioral confounders were smoking and alcohol drinking.

Physicians performed a general health assessment and physical examination, and blood samples were obtained at the field survey center. Blood samples were collected in the morning after 8 h of fasting, and all biochemical markers were analyzed on the same day. Glycated hemoglobin (HbA1c) was measured using ADVIA1650 Autoanalyzer, Bayer, MN, USA. Diabetes was determined if fasting plasma glucose > 126 mg/dL or HbA1c ≥ 6.5% or on diabetes medication. Hypertension was diagnosed if systolic blood pressure ≥ 140 mmHg or diastolic blood pressure ≥ 90 mmHg or on hypertension medication. Body mass index (BMI) was calculated as weight (kg) divided by the square of height (m^2^), and obesity was defined as a BMI of 25.0 kg/m^2^ or above, while the normal was BMI less than 25.0 kg/m^2^.

Oral examination, including denture status (dentate and denture), was performed by trained dentists.

### Statistical analysis

Differences in characteristics between cognitive impairment and non-cognitive impairment were compared using bivariate analyses such as T-test for continuous variables and chi-square test for categorical variables. Characteristic variables of the participants were described using frequency distributions for categorical variables and means with standard deviations for continuous variables. To compare the adjusted mean of SFR according to cognitive impairment, analysis of covariates (ANCOVA) was applied after controlling for various confounders.

Multivariable logistic regression analysis was used to evaluate the association between SFR and cognitive impairment after controlling for various confounders. The outcome was cognitive impairment, which was binary (no versus yes). Denture status, age, sex, education level, smoking, drinking, diabetes, hypertension, and obesity were considered as confounders since they were associated with cognitive function and/or salivary flow rate [15, 27]. Effect modification of sex and denture status were explored using stratified analysis, because previous studies [8, 28–30] reported the different association of masticatory function and tooth loss with cognitive impairment in sex and denture status. All analyses were performed using SPSS version 25.0 (SPSS, Inc., Chicago, IL).

## Results

Participants with cognitive impairment (*n* = 243) with lower total MMSE-KC score showed a higher prevalence of low SFR, higher age, and lower education but less smoking or drinking, hypertension, obesity than those without cognitive impairment (*n* = 406) (Table 1, Supplementary Table 1 and 2). There was no significant difference in denture status, sex, diabetes between participants with and without cognitive impairment.

**Table 1:** Characteristics of participants by cognitive impairment (*n* = 649)

Variable	*n*	Cognitive impairment		*P*-value
		No (***n*** = 406)	Yes (***n*** = 243)	
MMSE-KC score	649	27.0 ± 1.8	19.8 ± 3.3	< 0.001
Salivary flow rate (mL/min)				0.004
Normal (**≥** 0.3)	414	276 (68.0)	138 (56.8)	
Low (< 0.3)	235	130 (32.0)	105 (43.2)	
Hyposalivation (< 0.1)	97	66 (16.2)	31 (12.8)	
Denture status				0.2
Dentate	378	244 (60.1)	134 (55.1)	
Denture	271	162 (39.9)	109 (44.9)	
Age (year)	649	75.8 ± 5.2	76.8 ± 5.5	0.03
Sex				0.17
Male	211	140 (34.5)	71 (29.2)	
Female	438	266 (65.5)	172 (70.8)	
Education level				< 0.001
Junior school	495	287 (70.7)	208 (85.6)	
High school	154	119 (29.3)	35 (14.4)	
Smoking^a^				0.02
No	441	262 (64.5)	179 (73.7)	
Yes	208	144 (35.5)	64 (26.3)	
Drinking^b^				0.001
No	219	118 (29.1)	101 (41.6)	
Yes	430	288 (70.9)	142 (58.4)	
HbA_1_C	649	6.05 ± 0.8	6.12 ± 0.9	0.1
Diabetes^c^				0.09
No	451	292 (71.9)	159 (65.4)	
Yes	198	114 (28.1)	84 (34.6)	
Hypertension^d^				0.03
No	298	173 (42.6)	125 (51.4)	
Yes	351	233 (57.4)	118 (48.6)	
Obesity^**e**^				0.01
No	362	211 (52.0)	151 (62.1)	
Yes	287	195 (48.0)	92 (37.9)	

Data are presented as numbers (raw percentage) for categorical variables and mean ± standard deviation for continuous variables

*P*-values were obtained by Chi-square test for categorical variables and T-test for continuous variables

^a^Smoking: “No” refers to never smoked and “Yes” refers to past and current smoker

^b^Alcohol intake: No refers to never drunken, and “Yes” refers to past and current drinker

^c^Diabetes was determined as “Yes” if fasting plasma glucose > 126 mg/dL or HbA_1_C ≥ 6.5% or a history of diabetes

^d^Hypertension was determined as “Yes” if systolic blood pressure ≥ 140 mmHg or diastolic blood pressure ≥ 90 mmHg or taking hypertension medication

^e^Obesity: Body mass index (kg/m^2^) ≥ 25

MMSE-KC: Korean version of Mini-Mental State Examination in the Korean version of the Consortium to Establish a Registry for Alzheimer’s disease Assessment Packet (CERAD-K)

**Fig. 1:**
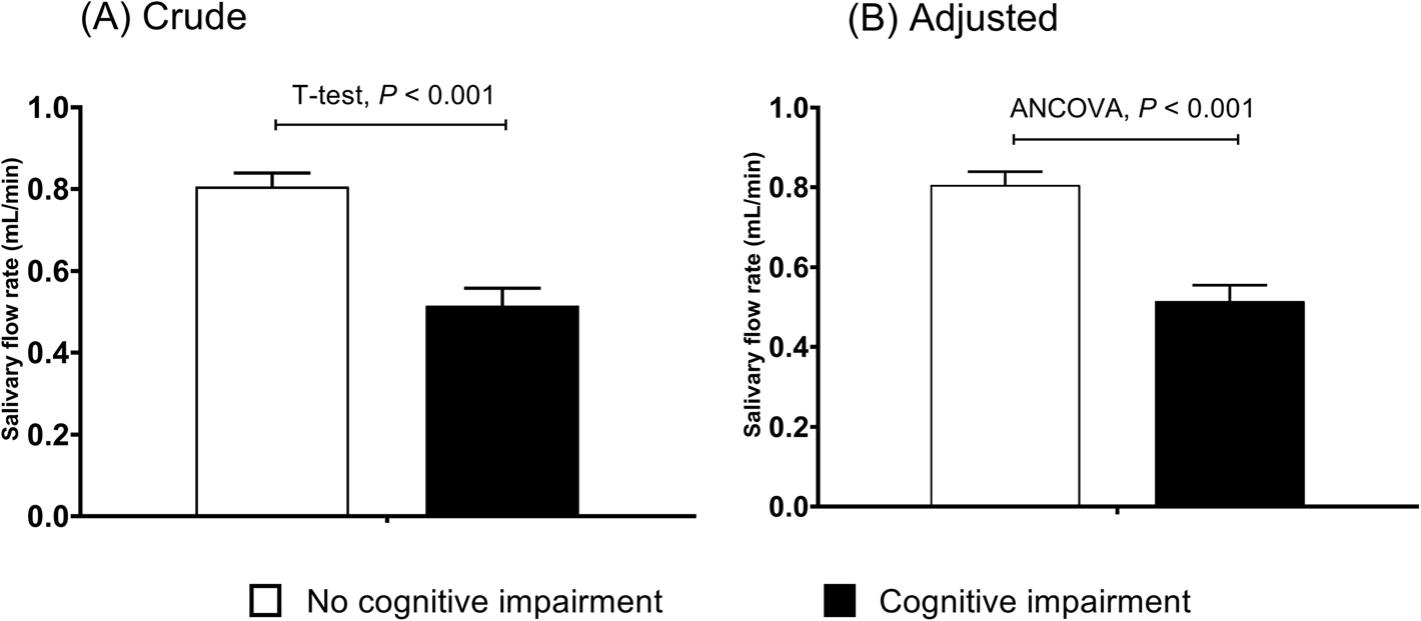
Salivary flow rate (mean ± SE) according to cognitive impairment (*n* = 649). **a** Crude (0.81 ± 0.04 for cognitive impairment controls versus 0.50 ± 0.03 for cognitive impairment cases); **b** Adjusted (0.81 ± 0.03 for cognitive impairment controls versus 0.52 ± 0.04 for cognitive impairment cases). Bar and whisker are mean and standard error. Adjusted values were from ANCOVA in the general linear model adjusted for denture status, age, sex, education level, smoking, drinking, diabetes, hypertension, and obesity

SFR was significantly higher by 1.6 times in both crude and adjusted value in participants without cognitive impairment compared with those with cognitive impairment (in crude, 0.81 ± 0.04 mL/min for non-cognitive impairment versus 0.50 ± 0.03 mL/min for cognitive impairment, *p <* 0.001; in adjusted, 0.81 ± 0.03 mL/min for non-cognitive impairment versus 0.52 ± 0.04 mL/min for cognitive impairment, *p <* 0.001) (Fig. 1).

Participants with low SFR (< 0.3 mL/min) were 1.5 times more likely to have cognitive impairment than those with normal SFR (odds ratio [OR] = 1.45, confidence interval [CI] = 1.05–2.11) (Table 2). Diabetes showed a significant association with a higher prevalence of cognitive impairment, while higher education, hypertension, and obesity showed a significant association with a lower prevalence of cognitive impairment.

**Table 2:** Adjusted association of salivary flow rate with cognitive impairment (*n* = 649)

Variables	OR (95% Confidence Interval)	*p*-value
Salivary flow rate		0.02
Normal (≥ 0.3 mL/min)	1	
Low (< 0.3 mL/min)	1.45 (1.05–2.11)	
Denture status		0.5
Dentate	1	
Denture	1.13 (0.80–1.61)	
Age (year)	1.01 (0.98–1.05)	0.4
Sex		0.4
Male	1	
Female	0.80 (0.50–1.31)	
Education level		< 0.001
Junior school or less	1	
High school or higher	0.43 (0.27–0.67)	
Smoking^a^		0.1
No	1	
Yes	0.67 (0.40–1.10)	
Drinking^b^		0.06
No	1	
Yes	0.71 (0.49–1.01)	
Diabetes^c^		0.02
No	1	
Yes	1.53 (1.07–2.20)	
Hypertension^d^		0.04
No	1	
Yes	0.69 (0.50–0.97)	
Obesity^e^		0.02
No	1	
Yes	0.66 (0.46–0.93)	

*p-*values were obtained by logistic regression adjusted for denture status, age, sex, education level, smoking, drinking, diabetes, hypertension, and obesity

^a^Smoking: “No” refers to never smoked and “Yes” refers to past and current smoker

^b^Alcohol intake: No refers to never drunken, and “Yes” refers to past and current drinker

^c^Diabetes was determined as “Yes” if fasting plasma glucose > 126 mg/dL or HbA_1_C ≥ 6.5% or history of diabetes

^d^Hypertension was determined as “Yes” if systolic blood pressure ≥ 140 mmHg or diastolic blood pressure ≥ 90 mmHg or taking hypertension medication

^e^Obesity: Body mass index (kg/m^2^) ≥ 25

**Fig. 2:**
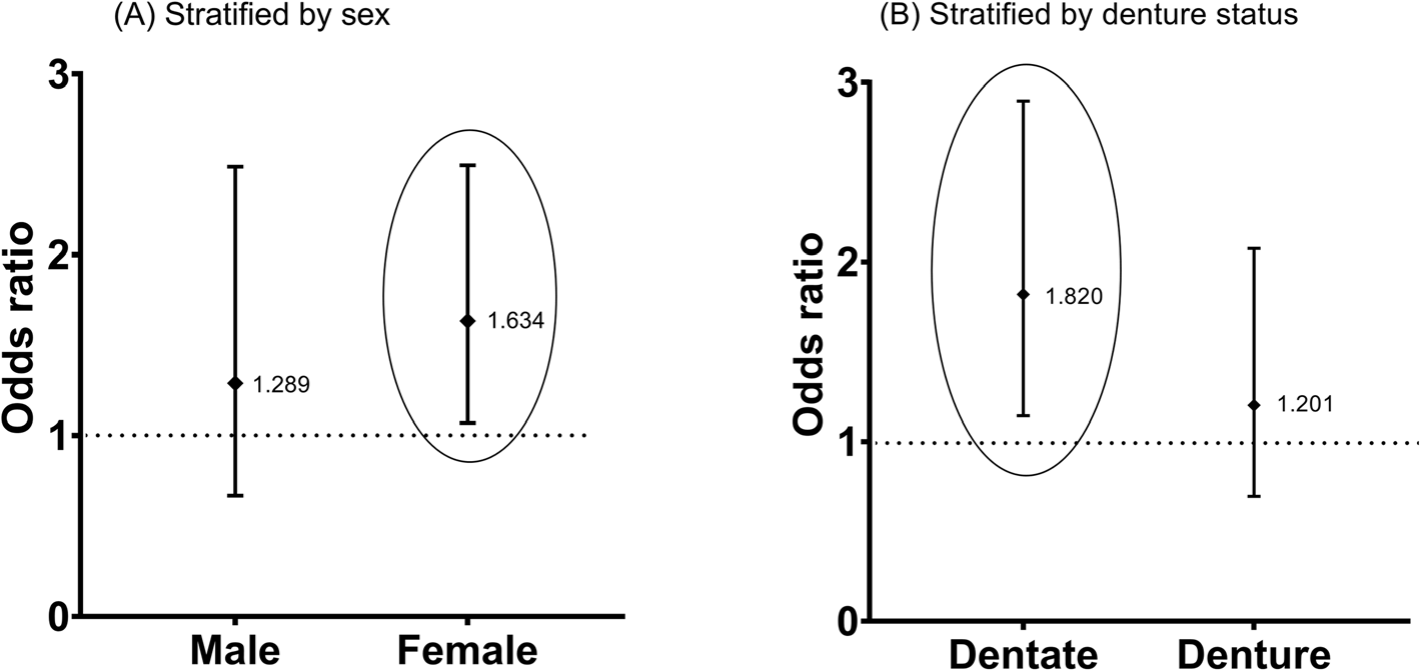
Sex- and denture status-stratified association of salivary flow rate (normal versus low) with cognitive impairment (*n* = 649). **a** Sex stratified: Male (Odds ratio [OR] = 1.29, Confident interval [CI]: 0.67–2.5, *P =* 0.45); Female (OR = 1.63, CI: 1.07–2.50, *p =* 0.02); **b** Dental status stratified: Dentate (OR = 1.82, CI: 1.14–2.90, *p =* 0.01); Denture (OR = 1.20, CI: 0.70–2.07, *p =* 0.51). OR were adjusted for denture status, age, sex, education level, smoking, drinking, diabetes, hypertension, and obesity, except for stratified variable in the multivariable logistic regression model. The diamond indicates the OR and a bar indicates 95% CI. The horizontal dotted line is the references as the null of association indicating the OR = 1

Stratified analyses by sex and denture status showed that the association between cognitive impairment and SFR was modified in females and participants with dentate (Fig. 2). In older women, the association of low SFR with cognitive impairment changed to OR of 1.63 (CI = 1.07–2.50), which was higher by 1.6 times compared with normal SFR. In dentate participants, the association of low SFR with cognitive impairment changed to OR of 1.82 (CI = 1.41–2.90), which was higher to 1.8 times compared with normal SFR. The association of low SFR with cognitive impairment was modified by about 10% higher in women (OR = 1.63 versus 1.50) and about 22% higher in dentate participants (OR = 1.82 versus 1.50).

## Discussion

This cross-sectional study showed that low unstimulated SFR was significantly associated with a higher prevalence of cognitive impairment adjusted for various confounders in Korean elders. The association was highly modified in women and dentate elders. To the best of our knowledge, this is the first study showing that low unstimulated SFR was independently associated with cognitive impairment after controlling for potential confounders, including denture status, socio-demographic factors, behavioral factors and general health problems.

The association between cognitive impairment and SFR was investigated previously. Ship et al. found the decline of submandibular gland function in people with early-stage dementia compared with healthy individuals [14]. The SFR was positively correlated to the cognitive level in Alzheimer patients, and their SFR decreased over time, opposing a stable direction in the controls [31]. The Danish study demonstrated that the prevalence of salivary gland hypofunction and daytime xerostomia was significantly higher in the cognitive decline group than in the non-cognitive decline group [13]. Our study confirmed the previous findings by demonstrating that elders without cognitive impairment had 1.6 times higher SFR level than those with cognitive impairment [13, 14], and elders with low SFR were more likely to have a risk of cognitive impairment prevalence by 1.5 times higher than those with normal SFR.

This study had four major strengths. Firstly, participants were recruited from the general resident population, not in a nursing home. Secondly, a medical professional evaluated cognitive impairment using the MMSE, the most widely used cognitive impairment screening tool in clinical practice and research. Thirdly, stratification analysis was performed to clarify the modification of the association. Fourthly, the association was adjusted for well-known potential confounders, including denture status, socio-demographic factors, behavioral factors and general health problems. Lastly, this study confirmed the previously reported significant association of diabetes mellitus [32] and education level with cognitive impairment [19]. Therefore, our study was valid enough to test the association of SFR with cognitive impairment.

Hitherto the mechanism of this relationship between cognitive impairment and SFR in human remains still unclear; some pathways on the relationship could be addressed. The salivary function is controlled by the autonomic nervous system and regulated by reflexes, including the afferent neural signal to the salivary centers in the brain and the efferent reflex [9, 33]. The chronic and progressive degeneration of the brain in cognitive impairment could alter the perception of the afferent impulses in the salivary centers leading to a decline in parasympathetic output, altering saliva production. Indeed, the downgraded activity of the cholinergic system was related to cognitive impairment [12]. However, this pathway could not change the stimulated SFR [13]. This may be due to the unstimulated SFR being more affected by the modulation of the salivary nuclei by a complex interaction with higher centers in the brain, including limbic and cortical centers [9, 34]. Recent reviews suggested that Alzheimer’s disease could affect the insular cortex leading to dysfunction of the autonomic nervous system [35, 36]. A Japanese study demonstrated that the stimulation of the posterior area of the insular cortex results in hyperactivity of both saliva and masticatory muscles in rats [37]. Thus, the cognitive impairment may dysregulate the salivary secretion through the autonomic nervous system modulated by the cortical network. Further studies are indicated to clarify the mechanism of this relationship in human.

In our study, the association between SFR and cognitive impairment was modified by sex and denture status. The association of low SFR with cognitive impairment increased by 10% in women, 22% in dentate participants, while the association in men and denture participants lost its significance (Fig. 2). Previous studies on cognitive impairment, dementia, and Alzheimer’s disease revealed a significantly higher prevalence and incidence rate in women than in men [28, 30, 38]. Besides, women showed a lower unstimulated SFR than men [39]. Therefore, the association between SFR and cognitive impairment could be increased in women. In contrast, our data showed a non-significant association in men, which was inconsistent with the result of the Danish study [13]. The reason may come from the differences in study design encompassing cognitive impairment assessment (MMSE for ours versus cognitive decline for Danish), age of the population (65 years or older for ours versus 56 for Danish), and the cut-off point for salivary flow rate (low SFR for ours versus hyposalivation for Danish). Although the sample size of hyposalivation (SFR (≤ 0.1 mL/min) in men with cognitive impairment (*n* = 6) and men without cognitive impairment (*n* = 17) was too small, our data also showed that hyposalivation was not associated with cognitive impairment in men. (Supplementary Table 3) Regarding the denture status, our data showed a higher prevalence of cognitive impairment in the denture group, which could mask the impact of low SFR on cognitive impairment in the denture group. Our results stratified by denture status supported the previous similar cognitive impairment studies that reported a stronger association of masticatory function with cognitive impairment in the dentate group than in the denture group [8, 29]. The mechanism behind the role of the denture in the association between SFR and cognitive impairment is still unknown.

Saliva is a unique fluid that contributes significantly to the maintenance of efficient chewing ability, swallowing activity [10, 40]. It also plays a vital role in digestive activity and modulation of microflora [41]. Thus, reduced SFR not only increases the risk of oral health problems [9] but also results in malnutrition due to masticatory difficulty [42]. People with cognitive impairment should be advised to use sugar-free chewing gum routinely and artificial saliva when needed and be monitored for oral fungal infection. As these patients are potential candidates for other oral health diseases [13, 43], aggressive preventive care, including daily care by family members or caregivers, and short-term regular oral health check-up by a dentist are recommended.

This study has some limitations. Firstly, due to the cross-sectional study design, the direction of causation cannot be inferred: whether SFR could be the outcome of cognitive impairment or SFR could influence the cognitive function. Secondly, the reproducibility of SFR was not tested. However, unstimulated saliva was collected for five minutes, which was appropriate according to the recommendation (1–6 min) from a previous study [44]. Lastly, anemia and the effect of the currently used medications related to increase of SFR such as zinc preparations and cholinesterase inhibitors were not considered in this study, which could draw our results a bit over-estimated. Future prospective cohort studies that include medication variables related to SFR and cognitive impairment, hypoglycemic agents, and specific antihypertensive medications will infer the causality and estimate unbiased association. Notwithstanding these limitations, our data was sufficient enough to meet the aim of this study.

## Conclusion

The low salivary flow rate was independently associated with cognitive impairment among Korean elders. The relationship was highly modified in females and dentate elders. Physicians and dentists should consider salivary flow rate and cognitive impairment as a risk factor between them simultaneously in clinics.

## Supplementary Information

**Additional file 1.**

**Additional file 2.**

**Additional file 3.**

## Data Availability

All data generated or analyzed during this study are included in this published article and its’ supplementary information files.

## Abbreviations

SFR: Salivary flow rate
MMSE: Mini-Mental State Exam
MMSE-KC: Mini-Mental State Exam Korean version
OR: Odd ratio
CI: Confidence interval
