# Supplementary material for: Salivary flow rate and the risk of cognitive impairment among Korean elders: a cross-sectional study
Source: BMC Geriatr. 2021 Apr 14;21:245. doi: 10.1186/s12877-021-02200-2 (PMC8045327; doi:10.1186/s12877-021-02200-2)
Supplement: Supplementary file 1 — Additional file 1. [file 12877_2021_2200_MOESM1_ESM.docx]

**SUPPLEMENTARY TABLE 1.** Item scores of MMSE-KC by salivary flow rate (n = 649)

| Variable | Salivary Flow Rate(ml/min) | | P-value |
| --- | --- | --- | --- |
|  | Normal(≥ 0.3)  (n = 414) | Low(< 0.3)  (n = 235) |  |
| Orientation in time (5 points) | 3.46 ± 1.04 | 3.16 ± 0.82 | < 0.001 |
| Orientation in place (5 points) | 3.79 ± 1.5 | 3.24 ± 1.70 | < 0.001 |
| Verbal memory (6 points) | 3.94 ± 1.33 | 3.62 ± 1.16 | 0.002 |
| Attention/calculation (5 points) | 2.39 ± 1.12 | 2.16 ± 0.81 | 0.003 |
| Language ( 5 points) | 5.07 ± 0.86 | 5.27 ± 0.78 | 0.004 |
| Praxis (3 points) | 2.19 ± 0.54 | 2.10 ± 0.47 | 0.01 |
| Visuospatial construction (1 point) | 0.63 ± 0.48 | 0.63 ± 0.49 | 0.94 |

Data are presented as mean± standard deviation. P-values were obtained by T-test.

MMSE-KC: Korean version of Mini-Mental State Examination in the Korean version of the Consortium to Establish a Registry for Alzheimer's disease Assessment Packet (CERAD-K)
