# Supplementary material for: Salivary flow rate and the risk of cognitive impairment among Korean elders: a cross-sectional study
Source: BMC Geriatr. 2021 Apr 14;21:245. doi: 10.1186/s12877-021-02200-2 (PMC8045327; doi:10.1186/s12877-021-02200-2)
Supplement: Supplementary file 2 — Additional file 2. [file 12877_2021_2200_MOESM2_ESM.docx]

**SUPPLEMENTARY TABLE 2.** Item scores of MMSE-KC by cognitive impairment (n = 649)

| Variable | Cognitive impairment | | P-value |
| --- | --- | --- | --- |
|  | No  (n = 406) | Yes  (n = 243) |  |
| Orientation in time (5 points) | 3.6 ± 1.0 | 2.9 ± 0.8 | < 0.001 |
| Orientation in place (5 points) | 4.1 ± 1.2 | 2.8 ± 1.8 | < 0.001 |
| Verbal memory (6 points) | 4.2 ± 1.2 | 3.2 ± 1.2 | < 0.001 |
| Attention/calculation (5 points) | 2.6 ± 1.1 | 1.8 ± 0.6 | < 0.001 |
| Language ( 5 points) | 5.2 ± 0.7 | 5.0 ± 1.0 | 0.003 |
| Praxis (3 points) | 2.2 ± 0.5 | 2.0 ± 0.4 | < 0.001 |
| Visuospatial construction (1 point) | 0.7 ± 0.5 | 0.5 ± 0.5 | < 0.001 |

Data are presented as mean± standard deviation. P-values were obtained by T-test.

MMSE-KC: Korean version of Mini-Mental State Examination in the Korean version of the Consortium to Establish a Registry for Alzheimer's disease Assessment Packet (CERAD-K)
