# Supplementary material for: Salivary flow rate and the risk of cognitive impairment among Korean elders: a cross-sectional study
Source: BMC Geriatr. 2021 Apr 14;21:245. doi: 10.1186/s12877-021-02200-2 (PMC8045327; doi:10.1186/s12877-021-02200-2)
Supplement: Supplementary file 3 — Additional file 3. [file 12877_2021_2200_MOESM3_ESM.docx]

SUPPLEMENTARY TABLE 3: Association of salivary flow rate with cognitive impairment in men (n=211)

| Variable |  | OR (95% Confidence Interval) | |
| --- | --- | --- | --- |
|  | N | Crude | Adjusted^*^ |
| Salivary flow rate(mL/min) |  |  |  |
| Normal (≥ 0.3) | 147 | 1 | 1 |
| Low (0.1 - 0.3) | 41 | 1.896 (0.936 - 3.841) | 1.777 (0.819 - 3.857) |
| Hyposalivation (<0.1) | 23 | 0.775 (0.287 - 2.094) | 0.709 (0.250 - 2.011) |

^*^Adjusted for denture status, age, sex, education level, smoking, drinking, diabetes, hypertension, and obesity
